# A Conversation with John Moon

**DOI:** 10.1021/acscentsci.3c01262

**Published:** 2023-10-24

**Authors:** Harini Bhat

In September, some major pharmacies in the U.S. began stocking over-the-counter naloxone, a nasal spray that reverses opioid overdoses sold under several brand names, including Narcan and RiVive. Although naloxone is now more accessible than ever, the story of how it moved from its original use in operating rooms in the 1960s to a spray that can be administered in the home remains largely unknown to the public.

**Figure:**
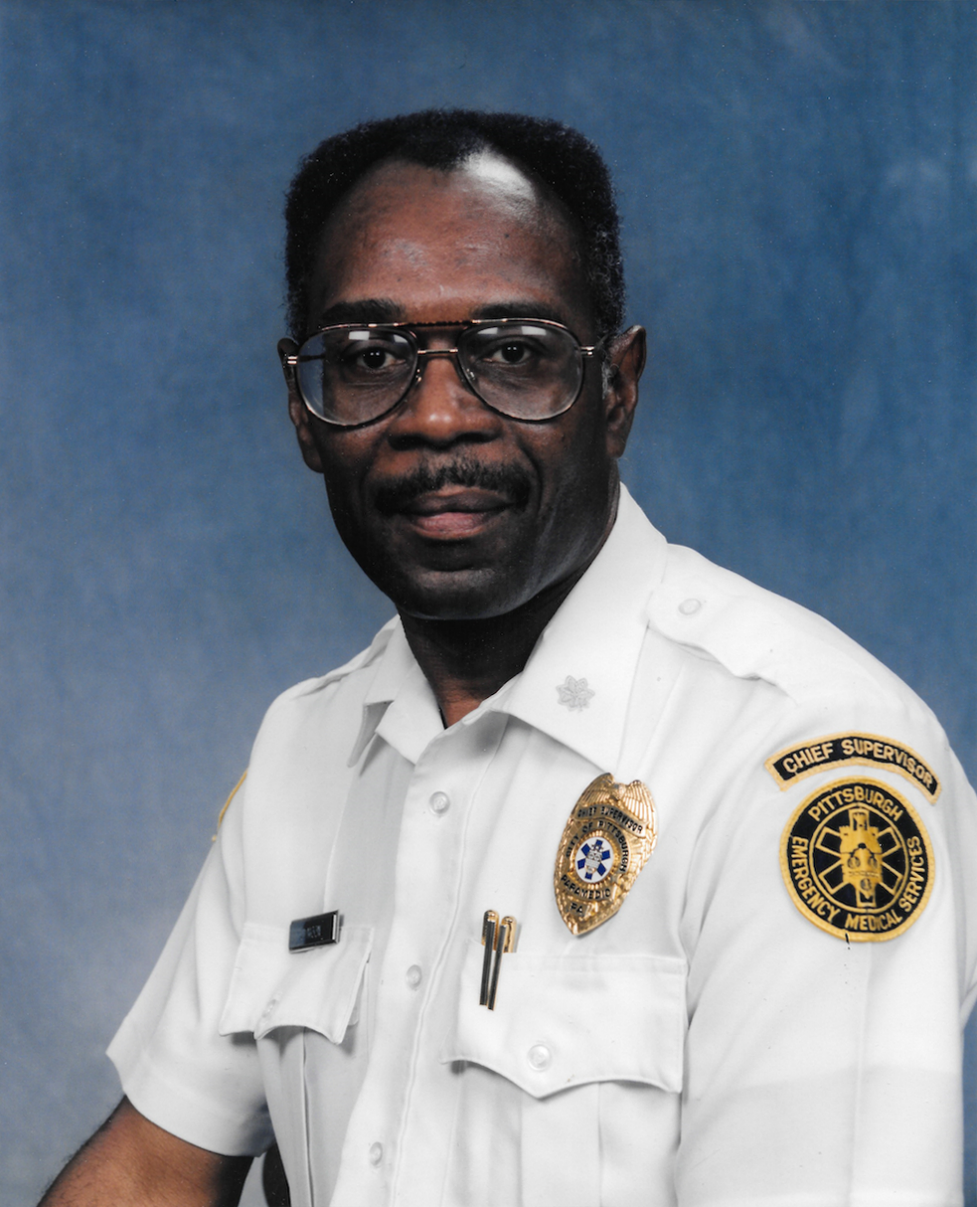
Moon working for Pittsburgh Emergency Medical Services in the 1980s. Credit: Courtesy of John Moon.

In the ’60s, hospital staff members used the newly discovered drug to alleviate side effects of opioid use, and in the ’70s they began giving it to patients after surgery to reverse opioid-involved anesthesia. But the drug was never used outside hospitals until the U.S.’s first all-Black paramedics team, the Freedom House Ambulance Service in Pittsburgh, developed and published protocols for using naloxone in the field.

Harini Bhat spoke with John Moon, a former Freedom House paramedic who eventually became assistant director of the Pittsburgh Bureau of Emergency Medical Services, about his firsthand experience administering naloxone starting in 1972. Moon discussed the health-care landscape before doctors recognized naloxone’s value in the field and talked about the paramedics who pioneered its use there to save patient lives. This interview was edited for length and clarity.

## What did Narcan look like back in the mid ’70s when you first administered it on the streets?

Definitely no Narcan nasal spray back then! We titrated naloxone into an IV bag of D5 [5% dextrose solution] and water and administered it that way. It was used for years in the operating room and the emergency room until Peter Safar, the founder of Freedom House Ambulance Service, put naloxone in our ambulance med kits to reverse opioid overdoses on the streets.

**Figure:**
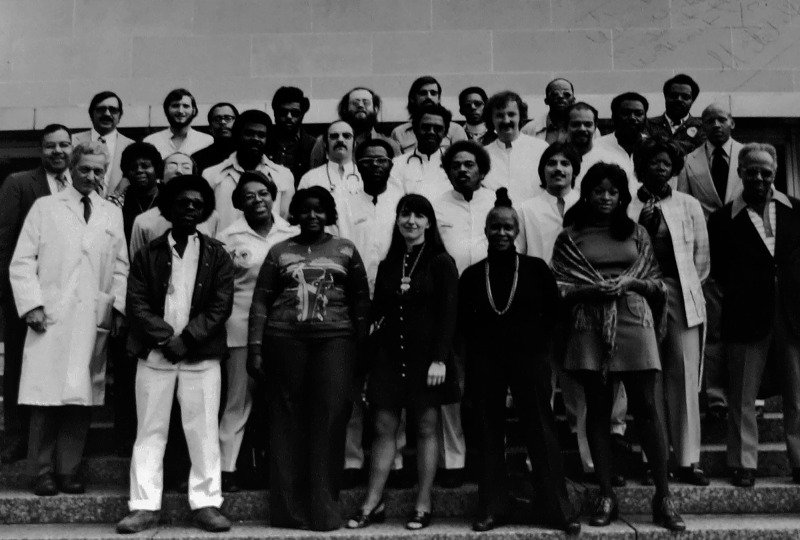
Freedom House Ambulance Service group photo. John Moon is in the second row, center, wearing glasses. Credit: University of Pittsburgh.

The general rule was to get the patient to the emergency room as fast as you possibly could. Basically that determined whether they lived or died. But, by redesigning the ambulances themselves and fully stocking them with medical equipment and medications, we made them hospitals on wheels. So that overall focus to bring naloxone to the patient—as opposed to the patient coming to the drug itself—was an extension of practices we had already been implementing.

I look at what we did back then and try to compare it to today with the OTC nasal spray formulation. We could control the naloxone dose through titration, which kept you in a relaxed state until we got you to a more controlled environment—the emergency room—unlike using a fixed-dose nasal spray, which can result in a more abrupt overdose reversal and withdrawal symptoms. That’s why it’s always important to call 911 and seek professional medical help after giving someone naloxone nasal spray. The spray is a short-term solution to a problem, and it’s greatly needed, but it does not replace in-hospital treatment.

## When did you get a sense that naloxone was a tool that was needed in the field?

When we first began implementing naloxone in the early ’70s in the Black communities of Pittsburgh, the rates of overdoses actually decreased there and increased in White neighborhoods. That’s when we knew this worked and began rolling it out across the country.

**Figure:**
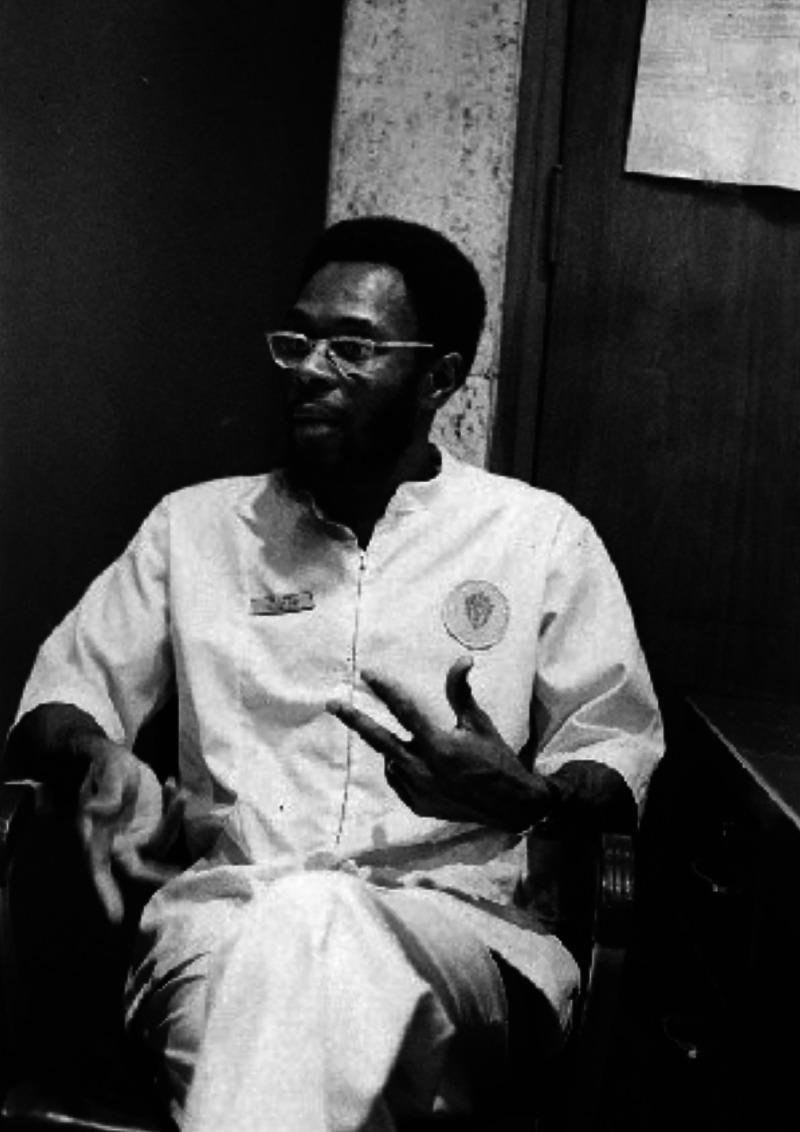
Moon in 1971, during his time as a paramedic for Freedom House. Credit: Courtesy of John Moon.

## How hard was it for paramedics and regular citizens to get naloxone in the ’60s and ’70s, and how did that change over time?

We were actually the only ones using it. No one else even conceived to take naloxone from one location to the next. We had to actually prove that the different procedures and techniques that you were using could be administered by a person that’s not trained to the level of a physician.

It wasn’t until Freedom House began writing the training manual for paramedics that paramedics began using naloxone across the country. [The manual was published in 1977.]

When the training program was implemented nationwide, administering naloxone became a standard practice among paramedics.

## How do you feel about the new OTC designation?

I’ve seen the change primarily as an improvement. I think it’s a very good idea to make it readily available to ordinary citizens.

Years ago, I was part of an organization called Prevention Point Pittsburgh that was a needle distribution site. In a perfect world [syringe service programs] would be something you would frown upon, but you have to look at the purpose behind it: to try to combat hepatitis or another AIDS epidemic.

Naloxone basically is the same way. You’re trying to combat a problem that is going to occur, regardless of whether naloxone is used or not. There will always be this risk as long as you have illicit drugs. In that regard, it’s a very good practice to make naloxone readily available to your ordinary citizen.

**Figure:**
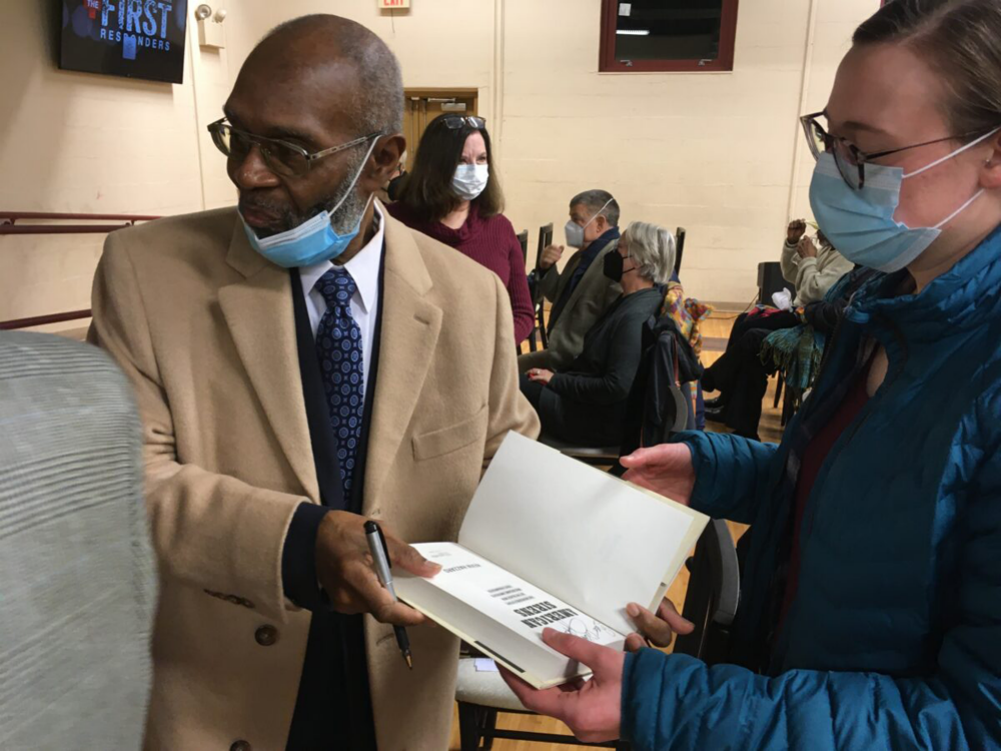
Moon signing a book in January at a showing of the WQED documentary *Freedom House Ambulance: The First Responders*. Credit: Bob Batz Jr./Pittsburgh Union Progress.

## What final words would you like to leave the readers with?

One of the things that we have to try to impress upon the general public is that a person who has recently received naloxone should still seek medical treatment. Naloxone has a short half-life inside the body, and the individual could unfortunately fall back into the overdose state.

Also, Narcan’s availability at the drugstore is a remarkable progression, but I want people to realize that Freedom House used naloxone to treat overdoses back in 1972. We were a group of Black men, part of a revolution to change the outlook on medical care in the community, much of which we take for granted today. It is not about John Moon so much as it is about the individuals that worked there and the organization itself. I’m just a vehicle that’s being utilized to get that point out there.

*Harini Bhat is a freelance contributor to*Chemical & Engineering News*, the independent news outlet of the American Chemical Society.*

*Recovery from addiction is possible. For help in the U.S., please call the free and confidential treatment referral hotline (1-800-662-HELP) or visit*findtreatment.gov. Visit the National Harm Reduction Coalition website to find naloxone*and other harm reduction resources near you. If you are in another country, call your local emergency hotline. You can find a directory of other helplines for addiction at*Helpguide.org.

